# A Clinical Approach to Brown Adipose Tissue in the Para-Aortic Area of the Human Thorax

**DOI:** 10.1371/journal.pone.0122594

**Published:** 2015-04-13

**Authors:** Huixing Wei, Seiichi Chiba, Chinatsu Moriwaki, Hirokazu Kitamura, Keisuke Ina, Taishi Aosa, Kenichiro Tomonari, Koro Gotoh, Takayuki Masaki, Isao Katsuragi, Hitoshi Noguchi, Tetsuya Kakuma, Kazuyuki Hamaguchi, Tatsuo Shimada, Yoshihisa Fujikura, Hirotaka Shibata

**Affiliations:** 1 Department of Endocrinology, Metabolism, Rheumatology and Nephrology, Faculty of Medicine, Oita University, 1–1 Idaigaoka Hasama, Yufu, Oita, Japan; 2 Department of Molecular Anatomy, Faculty of Medicine, Oita University, 1–1 Idaigaoka Hasama, Yufu, Oita, Japan; 3 Oita Diagnostic Imaging Center of Shonin Hospital, 9 Shoningahama, Bepu, Oita, Japan; 4 Noguchi Thyroid Clinic and Hospital Foundation, 7–52 Aoyama, Bepu, Oita, Japan; 5 Faculty of Medicine School of Nursing, Oita University, 1–1 Idaigaoka Hasama, Yufu, Oita, Japan; University of Minnesota, UNITED STATES

## Abstract

**Background:**

Human thoracic brown adipose tissue (BAT), composed of several subdivisions, is a well-known target organ of many clinical studies; however, the functional contribution of each part of human thoracic BAT remains unknown. The present study analyzed the significance of each part of human thoracic BAT in the association between regional distribution, cellularity, and factors involved in the functional regulation of thoracic BAT.

**Methods:**

We analyzed 1550 healthy adults who underwent medical check-ups by positron-emission tomography and computed tomography (PET–CT) imaging, 8 cadavers, and 78 autopsy cases in an observational study. We first characterized the difference between the mediastinum and the supraclavicular areas using counts of BAT detection and conditions based on PET–CT outcomes. The measurable important area was then subjected to systematic anatomical and immunohistochemical analyses using anti-uncoupling protein 1 (UCP1) antibody to characterize the cellularity in association with age and sex.

**Results:**

In PET–CT scanning, the main site of thoracic BAT was the mediastinum rather than the supraclavicular area (*P* < 0.05). Systemic macroanatomy revealed that the thumb-sized BAT in the posterior mediastinal descending para-aortic area (*pa*BAT) had feeding vessels from the posterior intercostal arteries and veins and sympathetic/parasympathetic innervation from trunks of the sympathetic and vagus nerves, respectively. Immunohistochemical analysis indicated that the *pa*BAT exhibited immunoreactivity for tyrosine hydroxylase and vesicular acetylcholine transporter located in the pericellular nervous fibers and intracellular UCP1. The brown adipose cells of *pa*BAT showed age-dependent decreases in UCP1 expression (*P* < 0.05), accompanied by a significant increase in vacuole formation, indicating fat accumulation (*P* < 0.05), from 10 to 37 years of age (*P* < 0.01).

**Conclusions:**

*pa*BAT may be one of the essential sites for clinical application in BAT study because of its visible anatomy with feeding vessels and sympathetic/parasympathetic innervation functionally affected by outer condition and senescence.

## Introduction

Brown adipose tissues (BATs) comprise brown adipose cells (BACs) expressing uncoupling proteins (UCPs) in mitochondrial inner membranes. UCPs of BACs drive the electrons derived from β-oxidation and the tricarboxylic acid (TCA) cycle not toward the biosynthesis of high-energy phosphate compounds but toward the generation of heat, contributing to surplus energy expenditure and excessive high-energy electron scavenging [1–5]. Progress in nuclear medicine, and specifically positron-emission tomography and computed tomography (PET–CT) scanning, has resulted in a new awareness that *Homo sapiens* also have BAT-regulating endothermal homeostasis, even in geriatric subjects [6]. Anatomically, human BACs have been identified in systemic areas in younger subjects and around large vessels in elderly subjects [7] and are classified into several subgroups, including multilocular, paucilocular, and unilocular types, according to the intracellular characteristics of the occupied pattern in the adipose vacuole area and the UCP1-containing cytosolic area [8–10].

It has been recently found that systemic white adipose cells and fibroblasts can be converted into brown adipose-like cells, beige/brite cells, by irisin, cold, catecholamines, and other stimuli [5,11,12]. Moreover, BACs can be interconverted into white adipose cells under warm conditions [13].

Although beige/bright cells may be derived from white adipose cells, classical BACs, derived from myoblasts and stem cells [14–17], are encapsulated by a collagenous membrane and have feeding vessels and innervation in the typical structure of an organ from the fetal period [14,16]. These structures of inherited BATs, composed of classical BACs, can be observed in areas along the path of the para-arterial/aortic region, and prominently around the thoracic aorta and renal and carotid arteries [18–25]. Recent evidence that para-arterial BATs can play an anti-atherosclerotic role in rodents [18] suggests that the inherited structural BATs around arteries is an antioxidative organ because of the confirmation of the scavenging system for excess oxidative electrons with the UCP family observed in animals from lower eukaryotic species through placental mammals [2].

Many recent breakthrough studies of human BATs using PET–CT analysis have targeted the supraclavicular area as the whole thoracic BATs (the sum of the two major parts: supraclavicular and mediastinal) and neck BATs and have characterized their physiological roles in homeostatic regulation [26–28]. However, among these important discoveries, we have seen little evidence describing the functional properties of each part of human thoracic BATs.

In the present study, we first investigated the characteristics of the two major parts of thoracic BATs to identify the macroanatomical sites that are critical in response to conditions, such as ambient temperature, sex, and age. Next, we analyzed the immunohistochemical properties of the critical site of thoracic BATs by sex and age with imaging techniques to determine the microanatomical characteristics of that part of the thoracic BATs.

## Materials and Methods

### Study Sample

This study followed institutional guidelines and was approved by the ethics committee of Oita University, Oita, Japan (No. 273, No. 385). It was a retrospective observational and case control study (UMIN CTR: UMIN000006066). All subjects (1550 healthy adults, 8 cadavers, and 78 autopsy subjects) provided written informed consent. For autopsy subjects under the age of 18, parents provided written informed consent. For analyzing glucose consumption, standardized uptake values of ^18^F-fluorodeoxyglucose (^18^F-FDG) were evaluated by PET–CT whole-body scans performed on 1550 healthy adults (858 males and 692 females) enrolled for cancer screening at Oita Diagnostic Imaging Center from July 2004 to June 2006 (Table 1). For the macrostructural study around the BAT-detected areas, a systemic anatomical dissection was performed to establish the innervation and feeding arteries and veins in four male and four female Japanese cadavers (61-, 63-, 65-, and 77year-old males and 77-, 79-, 89-, and 93-year–old females). To identify BATs in the samples, adipose tissues from the BAT-detected areas of 78 autopsy subjects were subjected to light microscop and electron microscopy to detect BACs (Table 2). To evaluate any difference between the UCP1-positive staining areas of BATs, samples from the thoracic descending para-aortic regions of 21 autopsy subjects were assessed with an immunohistochemical analysis of UCP1 (Table 2). None of the autopsy subjects had previously undergone PET–CT scanning.

**Table 1:** Classification of individual BAT detection areas in the thorax.

		All subjects						Male subjects						Female subjects				
Supraclaviculararea(*s*)	-	+		+		-	-	+		+		-	-	+		+		-
Mediastinalarea(*m*)	-	-		+		+	-	-		+		+	-	-		+		+
Class 1	*none*	*s − m*		*s + m*		*m—s*	*none*	*s − m*		*s + m*		*m—s*	*none*	*s − m*		*s + m*		*m—s*
Number	1494	7		29		20	843	1^a^		11		3^a^	651	6^a^		18		17^a^
Year of age	59 (52/68)	55 (50/64)		48 (42.5/55) * ^†^		54 (43/60.5)*	59 (52/68)	50 (50/50)		47 (37/55) *		38 (37/50) *	58 (51/67)	59.5 (50.5/67)		48 (42.8/56)* ^†^		57 (44.5/62.5)
Body mass index (BMI) (*kg/m* ^*2*^)	23.3 (20.9/25.6)	21.3 (17.3/24.4)		21.5 (19.3/23.1) *		21.3 (20.8/25.6)	24.1 (21.8/26.2)	16.5 (16.5/16.5)		22.1 (21.2/23.3)*		25.6 (20.4/26.9)	22.2 (20.3/24.5)	22.7 (19.1/24.9)		20.4 (18.4/22.5)*		21.1 (20.9/24.5)
Ambient temperature (*°C*)	17.5 (10.2/24.6)	24.1 (17/25.9)		7.4 (4.6/12.3)* ^†^		8.5 (4.78/12.4)* ^†^	17.9 (10.7/24.7)	15.2 (15.2/15.2)		5.9 (4.3/10.2)*		6.5 (4.2/8.5)*	17.3 (9.7/24.6)	24.9 (20.5/26.3)		10 (4.6/12.5)* ^†^		8.6 (4.9/14.1)* ^†^
Class 2	*none*		*s*			*m—s*	*none*		*s*			*m—s*	*none*		*s*			*m—s*
Number	1494		36^b^			20^b^	843		12^a^			3^a^	651		24^a^			17^a^
Year of age	59 (52/68)		50.5 (43/55.8) *			54 (43/60.5)*	59 (52/68)		48.5 (38.5/54^)^ *			38 (37/50)*	58 (51/67)		51.5 (43.8/59)*			57 (44.5/62.5)
Body mass index (BMI) (*kg/m* ^*2*^)	23.3 (20.9/25.6)		21.4 (19.3/23.5)*			21.3 (20.8/25.6)	24.1 (21.8/26.2)		21.9 (20.5/23.2) *			25.6 (20.4/26.9)	22.2 (20.3/24.5)		21 (18.6/23.8)*			21.1 (20.9/24.5)
Ambient temperature (*°C*)	17.5 (10.2/24.6)		10.2 (4.7/14.9)*			8.5 (4.7/12.4)*	17.9 (10.7/24.7)		6.1 (4.4/11.9) *			6.5 (4.2/8.5)*	17.3 (9.7/24.6)		12.1 (5.6/21.5) *			8.6 (4.9/14.1) * ^†^
Class 3	*none*	*s − m*			*m*		*none*	*s − m*			*m*		*none*	*s − m*			*m*	
Number	1494	7			49^b^		843	1^a^			14^a^		651	6^a^			35^a^	
Year of age	59 (52/68)	55 (50/64)			51 (43/57.5)*		59 (52/68)	50 (50/50)			45.5 (37/52) *		58 (51/67)	59.5 (50.5/67)			52 (43/59)*	
Body mass index (BMI) (*kg/m* ^*2*^)	23.3 (20.9/25.6)	21.3 (17.3/24.4)			21.5 (20.3/23.5)*		24.1 (21.8/26.2)	16.5 (16.5/16.5)			22.4 (21/24.1)		22.2 (20.3/24.5)	22.7 (19.1/24.9)			21.1 (19.5/23.4)	
Ambient temperature (*°C*)	17.5 (10.2/24.6)	24.1 (17/25.9)			8.5 (4.6/12.3)* ^†^		17.9 (10.7/24.7)	15.2 (15.2/15.2)			6.1 (4.3/8.9)*		17.3 (9.7/24.6)	24.9 (20.5/26.3)			9.7 (4.6/12.6) * ^†^	

Numerical data, except the number of subjects, are presented as medians (first quartile/third quartile). The column “All subjects” represents both sex populations, “Male subjects” represents the male population, and “Female subjects” represents the female population. The absence of BATs is represented by the symbol “-,” and the presence of BATs by “+.” The set *none* indicates the subject population, wherein BATs was not detected. The set *s* + *m* indicates the subject population, wherein BATs were simultaneously detected in the supraclavicular and the mediastinal areas. The set *s* − *m* indicates the subject population, wherein BATs were detected only in the supraclavicular area. The set *m* − *s* indicates the subject population, wherein BATs were detected only in the mediastinal area.

(*a*) The statistical difference between numbers in both sex *none* groups was <0.05 using Fisher’s exact test.

(*b*) The difference between numbers in both sex *BAT-detected* groups such as *s*, *m* − *s* of class 2 and *s* − *m*, *m* of class 3 was <0.05 using Fisher’s exact test.

* *p*-values referring to the same sex *none* group were <0.05 using the Wilcoxon’s rank sum test.

† *p*-values referring to the same sex “*s* − *m*” group were <0.05 using the Wilcoxon’s rank sum test.

**Table 2:** Age and sex distribution in the anatomical examination.

Years of age	0–10	11–20	21–30	31–40	41–50	51–60	61–70	71–80	81–90	All age
Male	14 (5)	7 (3)	7	1	5	1	5	5 (1)	1	46 (9)
Female	10 (3)	5 (1)	3 (1)	3 (1)	4 (2)	1	2 (1)	4 (3)	0	32 (12)
Both sex	24 (8)	12 (4)	10 (1)	4 (1)	9 (2)	2	7 (1)	9 (4)	1	78 (21)

Using hematoxylin and eosin (HE) staining, we examined 78 autopsied tissue samples followed by immunohistochemical staining of 21 samples using a human anti-UCP1 antibody. The numerical data correspond to HE staining and the numbers in parentheses to UCP1 immunohistochemical staining. In the histochemical examination, the age deviations (mean ± SD) were 30.9 ± 26.3, 30.9 ± 27.0 and 30.9 ± 25.7 for all 78 cases, 45 males, and 33 females, respectively. In the immunohistochemical study, the age deviations (mean ± SD) were 30.0 ± 28.4, 18.3 ± 22.9, and 40.7 ± 29.7 for all 21 cases, 9 males, and 11 females, respectively.

### Chemicals

To generate ^18^F-FDG (2-deoxy-2-[^18^F]fluoro-D-glucose) at a cyclotron facility, we used H_2_^18^O (Taiyo Nippon Sanso Corp., Tokyo, Japan) and mannose triflate (1,3,4,6-tetra-*O*-acethyl-trifluoromethanesulfonyl-beta-D-mannopyranose) (ABX GmbH, Radeberg, Germany). For light microscopic studies with routine immunohistochemistry, horseradish peroxidase (HRP; Dako, Kyoto, Japan), 3,3′-diaminobenzidine tetrahydrochloride (DAB; Dojindo, Wako, Kumamoto, Japan), bovine serum albumin (BSA; Sigma Chem, Perth, Australia), UCP1 (1/1000, Abcam Inc., Cambridge, UK), rabbit anti-human tyrosine hydroxylase (TH; 1/10000, Abcam Inc.), and rabbit anti-human vesicular acetylcholine transporter (vAChT; 1/10000, MBL, Nagoya, Japan) were used.

### Apparatus

We used the CYPRIS-HM10 cyclotron (Sumitomo Heavy Industries, Ltd., Tokyo, Japan) for ^18^F-FDG generation, a Discovery LS multidetector helical PET–CT scanner (GE Healthcare Japan, Tokyo, Japan) for PET–CT scanning, an Eclipse 50i (Nikon Co., Ltd., Tokyo, Japan) light microscope, a H-7650 (Hitachi Co., Ltd., Tokyo, Japan) transmission electron microscope, and a MacBook Pro (Apple Inc., CA, USA) for arithmetic processing.

### PET–CT scanning

We analyzed 3100 consecutive ^18^F-FDG PET–CT whole-body scans performed on 1550 healthy subjects (Table 1) for detect BATs robustly taking up ^18^F-FDG. Data of age, sex, height, and weight were obtained for all subjects. Outdoor temperatures in Oita on the dates of scans were obtained from the OITA Local Meteorological Observatory. Subjects were studied in the morning, from approximately 9 am to 12 noon, after an overnight fast beginning at 10 pm the night before. During the experiment, all subjects wore standardized clothing (0.49 clo, a unit of measure for the insulating properties of clothing). In a climate chamber, the subjects rested in the supine position under thermoneutral conditions (21°C) for 1 h. The ^18^F-FDG PET tracer was then intravenously administered, and scanning was performed 1 h after ^18^F-FDG administration. PET–CT scans were acquired using a multidetector helical PET–CT scanner and were analyzed using OpenPACS and PET–CT Viewer shareware. In areas where ^18^F-FDG uptake was identified by PET and the presence of fat was identified by CT, the maximal standardized uptake values (SUV_max_), defined as the activity per mL within the region of interest divided by the injected dose in megabecquerels per gram of body weight, and Hounsfield units (HU), a linear transformational scale as an arbitrary unit of X-ray attenuation, wherein water is assigned a value of zero and air is assigned a value of -1000, were determined. In the analysis of ^18^F-FDG PET–CT scans, BATs were considered present if tissue areas were >4 mm in diameter, had the CT density of adipose tissue (−150 to −30 HU), and had SUV_max_ of ^18^F-FDG of at least 2.5 g/mL in order to avoid the overestimation of the prevalence of activated BATs and the underestimation of malignancies [6,29,30].

### Classification of the BAT detection area

We first divided the thoracic areas of BAT detection into two groups, the area around the supraclavicular region (the supraclavicular, *s*), and the area around the posterior mediastinum (the mediastinal, *m*). According to the set theory and ISO 31–11 standard that defines mathematical signs and symbols for use in physical sciences and technology, these areas were indicated by five sets: set of *s*, set of *m*, intersection set of *s* and *m* (supraclavicular region with mediastinal; *s + m*), difference in set of *s* from *m* (supraclavicular region without mediastinal; *s − m*), and difference in set of *m* from *s* (mediastinal region with supraclavicular; *m—s*). These sets were then compared with other variables to identify correlations between these datasets. Several BAT-prone areas, such as external areas of the thorax and the pericardial area, were excluded from the study to avoid interference from extremely high-intensity signals of ^18^F-FDG uptake in the myocardium.

### Microanatomical examination

To confirm the presence of BACs and BATs, we examined 78 autopsies of Japanese subjects using histochemical/immunohistochemical methods and light and electron microscopy in the supraclavicular, thoracic para-aortal, axillary, subscapular, pericardial, para-renal hilar, and abdominal subcutaneous areas.

### Light microscopy

Immediately after removal, tissue pieces for light microscopy and immunohistochemistry were fixed overnight by immersion in 4% paraformaldehyde at 4°C. The next day, they were washed in 0.1 M phosphate buffer (PB, pH 7.4), dehydrated through an ethanol series, and embedded with paraffin wax. Sections for light microscopy were stained with hematoxylin and eosin.

### Immunohistochemistry

Sections were used for immunohistochemical analysis of UCP1, TH, and vAChT. Immunoreactivity was assessed according to the enzyme antibody technique with the following steps: endogenous peroxidase blocking with 3% hydrogen peroxide in methanol for 20 min at room temperature (RT), 5% normal goat serum in PBS (pH 7.2), 30 min at RT to block non-specific sites, and rabbit anti-human UCP1 (1/1000, Abcam Inc.), rabbit anti-human TH (1/10000, Abcam Inc.), or rabbit anti-human vAChT (1/10000, MBL, Nagoya, Japan) overnight at 4°C. The EnVision system with labeled polymer–HRP (30 min at RT) and DAB and 30% hydrogen peroxide in 0.05 M Tris-HCL buffer (pH 7.6, 10 min at RT) were used to identify positive signals.The sections were finally counterstained with hematoxylin.

### Electron microscopy

Immediately after removal, small tissue fragments were immersed in fixative comprising 2% glutaraldehyde and 2% formaldehyde in 0.1 M phosphate buffer pH 7.4, at 4°C for at least 4 h. The fragments were washed with PB and post-fixed in 1% osmium tetroxide, dehydrated in ethanol, and embedded in an Epon–Araldite mixture. Semi-thin sections (1 μm) were stained with toluidine blue. Further, 0.8-μm thin sections were obtained, stained with lead citrate, and examined by transmission electron microscopy.

### Digital image analysis of UCP1-positive and intracellular vacuole areas

Digital images were prepared for analyses using the ImageJ software (US National Institute of Health, Bethesda, MD, USA; http://rsb.info.nih.gov/ij/download.html). In each image, the region to be analyzed was selected and separated from the rest of the field using the image brush tool. The intracellular vacuolar area (no staining) and UCP1-positive area (brown staining) and total BAT area in the field analyzed were automatically measured using the color deconvolution plug-in. Color deconvolution involves the isolation of the color information from histological red, green, and blue (RGB) images containing multiple stains [31]. This was achieved by calculating the contribution of each stain based on the stain-specific RGB absorption. In this study, color deconvolution was used to isolate the DAB stain, indicating the UCP1-positive area, from the total area. Isolation from the whole area of the vacuole area, indicating the area of lipid droplets, also was performed by color deconvolution. Each image was transformed into 8-bit type (gray) then processed as binary (black and white) images. The scores for UCP1 expression and the intracellular vacuoles area in the analyzed field were calculated by dividing the target areas by the total BAT area.

### Quantative morphology of BACs and BAT

To characterize the morphological property of each BAT sample, we performed hierarchical cluster analysis, according to the outcome of preceding digital image analysis, wherein all BAT samples were assigned to at least three morphological subtypes: multilocular, paucilocular, and unilocular aspects of BACs. The 5-μm-thick sections stained for UCP1 were used to evaluate the morphology of the UCP1-positive adipose cells in each sample. Several morphological aspects were recorded, according to the form of the nucleus, intracellular vacuoles, and lipid droplets. The multilocular subtype revealed a round, centrally placed nucleus and a granular cytoplasm containing multiple fat vacuoles. The unilocular subtype demonstrated a crescent-shaped, marginally placed nucleus and a granular cytoplasm containing a large fat vacuole. Furthermore, the paucilocular subtype showed an intermediate-shaped nucleus and a granular cytoplasm containing an intermediate morphology of fat vacuoles between multilocular and unilocular cells [7,8,32]. We used Ward’s hierarchical clustering analysis to assign the results of immunohistochemistry by the percent of area occupied with the multilocular, paucilocular and unilocular cellularities into three subgroups: multilocular-dominant, paucilocular-dominant, and unilocular-dominant group, respectively. Hierarchical clustering analysis, an attempt to identify homogeneous subgroups of cases as reported by Eisen et al. [33], reassigns every data point to a subgroup by Ward’s minimum variance method calculated the combination that a sum of squares is minimized of Euclidean space distance between each data and shows the clustering result in a dendrogram with a heat map representing normalized distances of individual samples, using a statistical software (JMP 11.0, SAS Institute, Cary, NC, USA). We determined the morphology of these three subtypes of BAT samples by their histological aspects, automatically calculated their histological properties with imaging software, and confirmed the assignments of all samples to three morphological subgroups: multilocular-dominant (multilocular: multi), paucilocular-dominant (paucilocular: pauci), and unilocular-dominant (unilocular: uni). Next, by univariate multinomial logistic regression, we assessed whether sex and age were contributors to BAC morphology. We reassigned these three groups to three sets: multilocular vs. other, paucilocular vs. other, and unilocular vs. other. We finally evaluated how sex and age contributed toward determining the BAC morphology and calculated the critical threshold of the parameter by univariate binomial logistic regression.

### Statistical Analysis

Statistics are expressed as median (first quartile/third quartile). Continuous variables and incidence values were compared by the Wilcoxon’s rank sum test and Fisher’s exact probability test, respectively. All samples were assigned and multivariate comparison of normally distributed continuous variables was performed by hierarchical clustering and analysis of covariance (ANCOVA), respectively. The contribution of independent variables to the properties of dependent variables and the evaluation of adequacy between several models were verified by multivariate multinomial logistic regression analysis. The area under the curve (AUC), sensitivity, specificity, and cut-off value were also calculated in integrated logistic models. Statistical significance was set at *P* < 0.05. All calculations were performed with SAS software, JMP 11, except for Fisher’s exact probability test of hypercomplex contingency tables using SPSS statistics (version 17, IBM Corp, NY, USA).

## Results

### Prevalence of ^18^F-FDG-accumulating BATs in thorax with PET–CT at medical check-up

We detected 56 (3.6%) cases with ^18^F-FDG-accumulating BATs in 1550 healthy adults. The cases with ^18^F-FDG-accumulating BATs comprised 15 males and 41 females; the number of females was significantly higher (Fisher’s exact probability test; *P* < 0.0001). We investigated the BAT-detected areas and confirmed that these areas comprised two major subareas: supraclavicular (*s*) and posterior mediastinal (*m*). Because the 56 cases of BAT detection clearly showed several patterns of distribution of BAT-detected subareas, including *s − m*, *s + m*, *m − s*, *s* and *m*, we classified these subareas into three classes, besides the set *none* indicating the population of 1496 subjects, wherein BATs was not detected (Table 1). Table 1 shows three classes: sets of *s*, *s + m*, and *m* (class 1), sets of *s − m* and *m* (class 2), and sets of *s* and *m − s* (class 3). The prevalence of females was significantly higher than males in sets *s − m* and *m − s* of class 1, *s* and *m − s* of class 2, and *s − m* and *m* of class 3. A significantly lower age was observed compared with *none* in the sets of *s + m* and *m—s* of all and male subjects, *s + m* of female subjects of class 1, in the sets of *s* and *m—s* for all and male subjects, *s* of female subjects in class 2, in the sets of *m* for all, male, and female subjects of class 3. A significantly lower age was observed, compared with *s − m*, in the sets of *s + m* of all and female subjects in class 1. Moreover, a significantly lower BMI was detected compared with *none* in the sets of *s + m* for all, male, and female subjects in class 1; in the sets of *s* for all, male, and female subjects in class 2; and in the set of *m* for all subjects in class 3. A significantly lower ambient temperature was found compared with *none* in the sets of *s + m* and *m − s* for all, male, and female subjects in class 1; in the sets of *s* and *m − s* for all, male, and female subjects in class 2; in the sets of *m* for all, male, and female subjects in class 3. A significantly lower ambient temperature was found compared with *s − m* in the sets of *s + m* and *m − s* for all and female subjects in class 1 and in the sets of *m* for all and female subjects in class 3. We compared the prevalence of each subarea by class to find which subarea was the major area and found that the subarea *m* in class 3 (7/49) had a significantly larger population than the subarea *s* of class 2 (36/20) by Fisher’s exact test (*P* < 0.05).

### Anatomical examination

**Fig 1:**
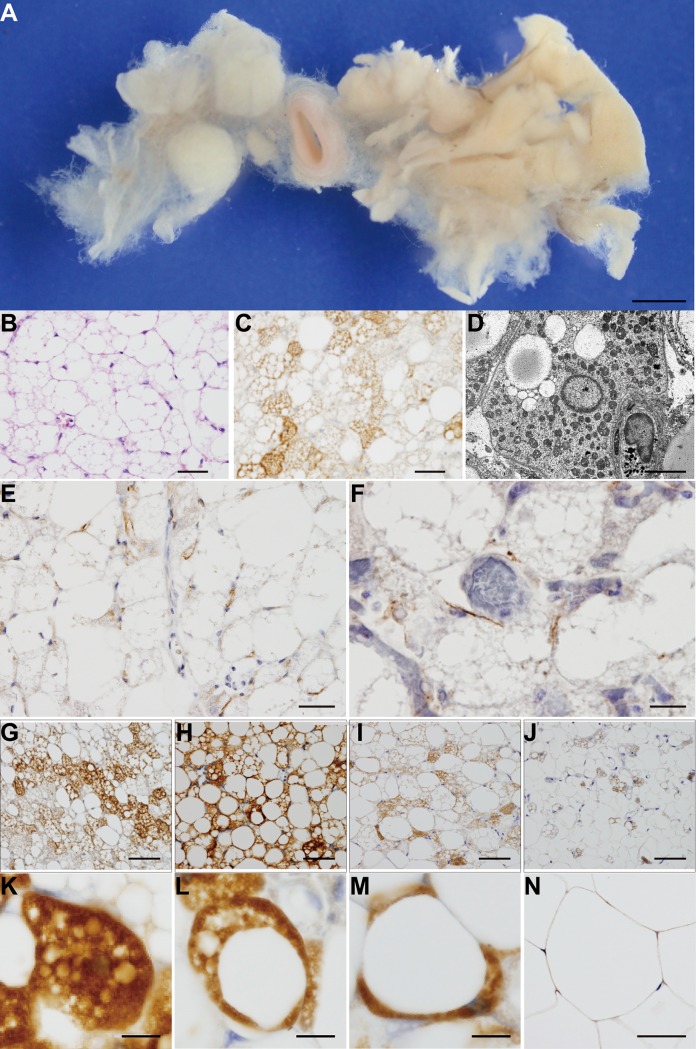
Overview of BATs and cells. (A) Typical BATs in the thoracic periaortic area of a 16-year-old male. (B) HE staining, (C) UCP1 immunohistochemical staining and (D) transmission electron microscopy. Immunohistochemical staining for TH (E) and vAChT (F). Morphological properties of UCP1 immunohistochemical staining of BATs for the multilocular (K), paucilocular (L), and unilocular (M) types. (G) Multilocular-predominant, (H) paucilocular-predominant, (I) paucilocular-predominant, (J) unilocular-predominant. (N) The unilocular adipocytes of subcutaneous white adipose tissue. Scale bars: (A) 10 mm, (B)(C)(E)(F)(G)(H)(I)(J)(N) 50 μm, (D) 5 μm, and (K)(L)(M) 10 μm.

Fig 1A shows the typical BATs in the mediastinal descending para-aortic area of a 16-year-old male in the macro-anatomical study. The brown adipose soft tissues around the left side of the descending thoracic aorta exhibited the classical aspects of BATs accompanied by abundant vessels (Fig 1). These organs were located around the space between the thoracic descending aorta and the thoracic vertebrae, dominantly on the left side, with a yellow-brownish aspect, under the parietal pleura and in front of the periosteum of the vertebrae, surrounded by numerous collagen fibers and penetrated by many small vessels and innervations. We confirmed that these vessels originated from the posterior intercostal arteries and branched from the thoracic aorta and that the posterior intercostal veins drained to the azygos veins. Sympathetic bundles branching from the sympathetic nerve trunk and parasympathetic fine fibers from the left vagus nerve had penetrated BATs, along the courses of the vessels feeding BATs. These vessels and fine nervous fibers were feeding the para-aortic and intercostal BATs from near the costotransverse joints up to the intercostal areas.

**Fig 2:**
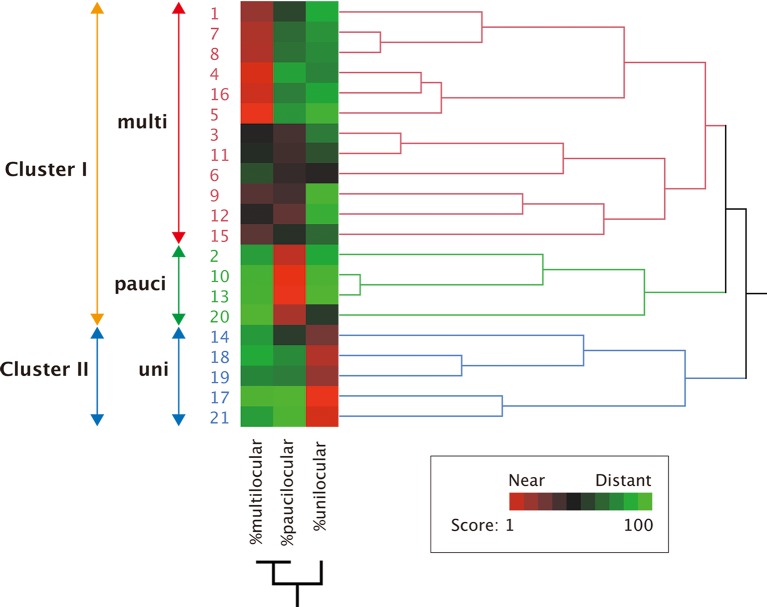
Hierarchical clustering according to BAC cellularity. Summary of hierarchical clustering analysis of the immunohistochemical data of 21 UCP1-positive cases. The number and the word represent the individual case and each cellularity, respectively. The arrows show the range of the same cluster. Further, the branch length represents the similarity between results obtained in this system. UCP1-positive cases in the present study were classified into three groups according to the results: multi, the multilocular-dominant group; pauci, the paucilocular-dominant group; uni, the unilocular-dominant group. Cluster I comprised multi and pauci, Cluster II was equal to uni. %multilocular, percentage of area occupied with the multilocular cells; %paucilocular, percentage of area occupied with the paucilocular cells; %unilocular, percentage of area occupied with the unilocular cells. The score of the heat map in the lower right box represents the normalized distance among parameters.

Following the outcome in the macro-anatomical study, we investigated this area in 78 autopsy cases by microscopy using histochemical techniques (Table 2). In all the autopsy cases, we detected several clusters of cells with a typical multilocular appearance (Fig 1B and 1D) or with cells staining positively for UCP1 by immunohistochemical techniques (Fig 1C and 1G–1M). Few BACs were found in the white adipose tissue obtained from the abdominal subcutaneous region (Fig 1N). We then investigated the expression of TH and vAChT, strongly associated with sympathetic and parasympathetic nerves, respectively, and revealed the positive brown staining of TH (Fig 1E) and vAChT (Fig 1F) in BATs. We also observed variation in the extent of UCP1 staining of BATs in the 21 cases (Table 2). The age (mean ± SD) was 30.0 ± 28.4, 18.3 ± 22.9, and 40.7 ± 29.7 for the 21 cases, 9 males, and 11 females, respectively. Using ImageJ, the values of % UCP1-positive staining area were 18.5 ± 9.8, 21.9 ± 7.8, and 15.4 ± 10.8 for all 21 cases, 9 males, and 11 females, respectively. Further, the outcomes for % vacuole area were 75.0 ± 11.5, 70.7 ± 10.2, and 78.9 ± 11.6, respectively. These two parameters showed no statistically significant sex difference. Moreover, we observed diversity in intracellular aspects (Fig 1G–1J), wherein the multilocular type of BAC demonstrated highly UCP1 antibody-stained cytosol with many small vacuoles (Fig 1K); the paucilocular type had an intensely UCP1 antibody-stained cytosol with many small and few larger vacuoles (Fig 1L) and the unilocular type showed poorly UCP1 antibody-stained cytosol with large vacuoles (Fig 1M) that was clearly distinct from the unilocular type of white adipose cells with mild UCP1 antibody staining (Fig 1N). Because of the diversity of the intracellular aspects (Fig 1G–1M), we classified the 21 cases according to UCP1 staining and vacuoles into three locular types: multilocular, paucilocular, and unilocular cells and assigned all samples into clusters based on the calculated percent area occupied by each cellular type. Hierarchical clustering was performed to assign the results of UCP1-positive samples and the correlations were graphically displayed with JMP 11 (Fig 2). The patterns of locularity were nearly identical in terms of staining in the majority of UCP1-positive samples, particularly in the patterns of the multilocular and the paucilocular cells. In contrast, there were less similar scoring patterns among the unilocular and multilocular cells, suggesting that the dominant pattern of locularity of UCP1-positive sample correlated with the individual difference. The results confirmed the classification of the 21 UCP1-positive cases into two clusters: cluster I (16 cases) and cluster II, the unilocular-dominant group (uni) (5 cases). Cluster I was further subclassified into multilocular-dominant (multi) (12 cases) and paucilocular-dominant (pauci) (4 cases) according to the branch length, representing the correlation of the scoring data (Fig 2). The 21 UCP1-positive cases were finally assigned to three subgroups: multi, pauci, and uni (Fig 2).

**Fig 3:**
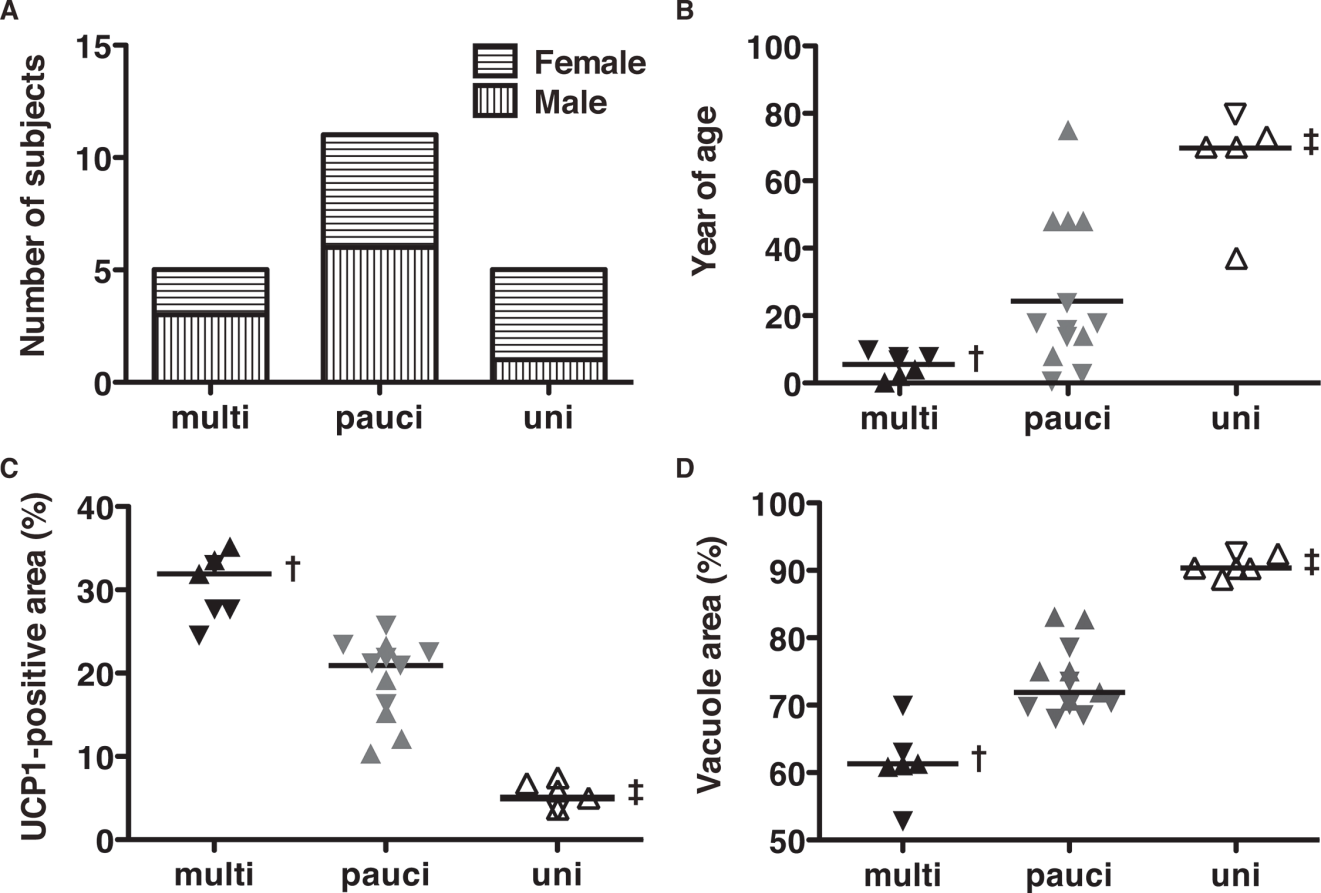
Determinants of BAT morphology. We evaluated the ratio of UCP1-positive and vacuole areas associated with sex, age, and BAT morphology using ImageJ. The inverted triangles and triangles represent males and females, respectively. The solid, gray, and open symbols represent multilocular (multi), paucilocular (pauci) and unilocular (uni), respectively. The flat bar of each group in B, C, and D indicates the median value of each group. (A) Sex distribution of each BAT morphology. (B) Age distribution of each BAT morphology. (C) UCP1-positive area (%) distribution of each BAT morphology. (D) Vacuole area (%) distribution of each BAT morphology. The symbols † and ‡ represent p < 0.05 referring to the paucilocular and unilocular types and the unilocular type, respectively. The symbol ‡ represents p < 0.05 referring to the paucilocular type.

We investigated whether the locular aspects were affected by sex, age, % UCP1-positive area, or % vacuole area using Fisher’s exact probability test and Wilcoxon's rank sum test followed by Bonferroni correction. The numbers of male and female subjects were 3and 2 in the multilocular type (multi), 6 and 5 in the paucilocular (pauci), and 1 and 4 in the unilocular (uni) BAT morphology. As shown in Fig 3A, 3B, 3C and 3D, the type—multilocular (multi), paucilocular (pauci), or unilocular (uni)—showed significant differences in % UCP1-positive area, % vacuole area, and age; however, there was no sex difference. These differences were particularly observed in the unilocular type.

Table 3 shows that the odds ratios of the multilocular and paucilocular types compared to the unilocular type significantly decreased by aging but not by sex difference and that the odds ratio of multilocular versus paucilocular showed less significance by age or sex difference (Table 3). Because the effect of the number of events per variable (EPV) was less than 10 (21 events, 3 variables) [34], we rearranged the three classes of locularity into two classes, a locular type vs. other, and re-evaluated the odds ratio for each class by univariate logistic analysis. Aging significantly modulated the odds ratios, which decreased in the multilocular vs. other class and increased in the unilocular versus other class (Table 4). The specificity and sensitivity for the multilocular type vs. other morphological types were 81.25% and 81.25% and those for the unilocular type were 81.25% and 81.25%, respectively. There was no significant sex difference in any class or by aging in the paucilocular vs other.

**Table 3:** Univariate multinomial logistic regression analysis of BAT morphology.

			Univariate multinomial logistic models		
	uni/uni	multi/uni	pauci/uni	multi/pauci	
Variable	Odds Ratio	Odds Ratio (95% CI): p	Odds Ratio (95% CI): p	Odds Ratio (95% CI):p	p value
Sex (M vs F)	1	0.41 (0.07–1.53): 0.2145	0.46 (0.09–1.42): 0.2173	0.89 (0.28–2.62): 0.8386	0.3375
Year of age	1	0.78 (0.58–0.92): 0.034*	0.93 (0.84–0.98): 0.0311*	0.84 (0.63–0.98): 0.1227	0.0001^†^

The odds ratio and 95% confidence interval (95% CI) of sex and age were estimated according to the morphology of descending para-aortic BATs. The column uni/uni represents analysis of the unilocular type referring to the unilocular; multi/uni, multilocular type referring to the unilocular type; pauci/uni, the paucilocular type referring to the unilocular type; and multi/pauci, the multilocular type referring to the paucilocular type. Odds ratios (95% CI): associated *p*-values are shown in the univariate column, except the odds ratio of uni/uni, which is represented as 1.00. The outcome of univariate multinomial logistic analysis for each variable is shown in column *p*.

* *p*-values refer to the unilocular type of every variable as <0.05.

† *p*-values of the univariate multinomial logistic analysis of every variable were <0.05.

**Table 4:** Univariate logistic regression analysis of BAT morphology.

		Multilocular vs others			Paucilocular vs others			Unilocular vs others	
Variable	Odds Ratio (95% CI)		p value	Odds Ratio (95% CI)		p value	Odds Ratio (95% CI)		p value
Sex (Male vs. Female)	0.52 (0.06–3.98)		0.5248	0.56 (0.09–3.11)		0.5042	5.14 (0.59–113.4)		0.1444
Years of age	0.84 (0.63–0.97)		0.0030^†^	1.02 (0.98–1.05)		0.3489	1.09 (1.03–1.19)		0.0006^†^

The odds ratio, 95% confidence interval (95% CI) and associated p-values of sex and age were estimated according to the morphology of the descending para-aortic BATs. The column *Multilocular vs*. *others* represents analysis of the multilocular type referring to the other locular types; *Paucilocular vs*. *others*, of the paucilocular type referring to the other types; *Unilocular vs*. *others* of the unilocular type referring to the other types, respectively.

† *p*-values of the univariate logistic analysis of the variables were <0.001.

**Fig 4:**
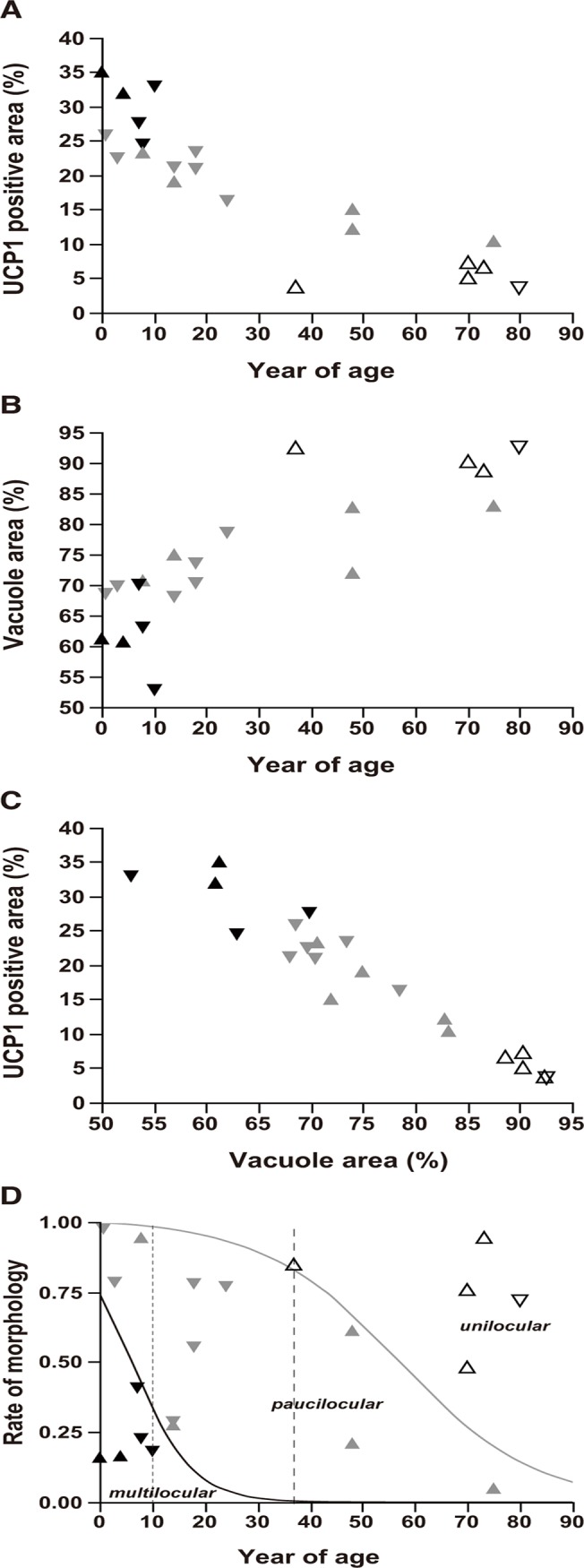
Aging and BAT morphology. Inverted triangle and triangle symbols represent males and females, respectively. The solid, gray, and open symbols indicate the multilocular (multi), paucilocular (pauci), and unilocular (uni) types, respectively. (A) Scatter plot of UCP1-positive area (%) *vs*. age. (B) Scatter plot of vacuole area (%) *vs*. age. (C) Scatter plot of UCP1-positive area (%) *vs*. vacuole area (%). (D) Sigmoid plot of morphological proportion vs. age using logistic regression. The morphological proportion represents the proportion of occupation of each morphological type compared to the other types. The solid and gray curves indicate the morphological proportion of the multi and uni types, respectively. The left lower, middle, and right upper regions show the distribution of the multi, pauci, and uni types of BAT morphology. The fine and rough broken lines show the age cut-off values of 10 years for the multi and 37 years for the uni types, respectively.

Using ANCOVA, we revealed little contribution of sex, adjusted for age, to the distribution of percentage of UCP1-positive (Fig 4A) or vacuole areas (Fig 4B). The sex-adjusted percentage of vacuole had a significant negative correlation with the percentage of UCP1-positive area (*r* ^*2*^ = 0.93, RMSE = 2.83, *p* < 0.0001) (Fig 4C), whereas no contribution of sex was observed. Sex-unadjusted aging had a significant negative correlation with % UCP1-positive area (*p* < 0.0001, *r* ^2^ = 0.78) and positive correlation with % vacuole area (*p* < 0.0001, *r* ^2^ = 0.70). Furthermore, the % UCP1-positive area had a significant negative correlation with % vacuole area (*p* < 0.0001, *r* ^2^ = 0.97). However, no significant sex difference was observed.

Because the significance of the contribution of age to the morphology of BATs was confirmed in the logistic analysis (Table 4), we reveal in Fig 4D a logistic plot, showing the performance of two prediction models for the morphology of BATs, predominantly in the multilocular (solid line) and unilocular types (gray line). The prediction model for multilocular predominancy of BATs showed that AUC for the multilocular predominant type was 0.88 (cut-off value of age = 10.0 years) and that for the unilocular predominant type was 0.93 (cut-off value of age = 37 years). The prediction model for the paucilocular predominancy of BATs with little significance (Table 4) showed that AUC and the cut-off value for age were 0.53 and 48.0 years (ROC curve not shown). These anatomical outcomes suggested that senescence significantly affected the fraction of the UCP1-positive or vacuole areas and has a marked effect on the locularity of BAC.

## Discussion

Outcomes of our PET–CT examination were compatible those of with previous studies in the prevalence of thoracic BAT detection independently facilitated by sex, age, BMI, and ambient temperature among the population, particularly in the supraclavicular area of class 2, a supraclavicular-dominant classification (Table 1) [35–37]. However, the mediastinal area of class 3, a mediastinal-dominant classification, was greatest among all subclasses of every classification. Because the measurable amount of BATs in the mediastinal area, even considering the lower significance of BMI in BAT detection, was consistent with previous reports [8,22,36,38], we focused on this area for further anatomical examination. Fig 1 shows a general overview of BATs and BACs. These typical BACs (Fig 1B–1D) were found in 78 autopsy samples from the descending para-aortic region in the thorax (Fig 1A) (Table 2) succeeded to ascertain the UCP1-positive staining (Fig 1G–1M), completely different from that in white adipose cells (Fig 1N), in 21 autopsy samples (Table 2). We also found positive vAChT staining in the pericellular area of typical para-aortic BACs (Fig 1F) in addition to the pericellular positive staining of TH (Fig 1E). This evidence of biphasic autonomic innervations around human BACs was a novel observation in human anatomy and compatible with those of previous studies in rodents [39–41]. The systemic dissection of eight cadavers clearly confirmed the autonomic double innervation of para-aortic BATs by left vagus nerves and left sympathetic nerve trunk along with the posterior intercostal arteries branching from the descending thoracic aorta and the proximal location of para-aortic BATs to intercostal BATs in the courses of feeding arteries.

Because samples from this organ revealed a histologically distinctive aspect (Fig 1G–1J) comprising three types of BACs, multilocular (Fig 1K), paucilocular (Fig 1L), and unilocular (Fig 1M), patterns [7,9,42,43] with diversity of the area of UCP1-staining and multishaped vacuole and lipid droplets by hierarchial cluster analysis (Fig 2), affected by age rather than sex (Fig 3A, 3B, 3C and 3D), we performed an univariate logistic analysis and confirmed the significant contribution of age to the determination of BAT morphology (Table 3). We then found the cut-off age of BAT morphology, wherein that of the multilocular pattern was under 10 years old and of the unilocular pattern was over 37 years old (Fig 3) (Table 4). The low significance of age for the paucilocular pattern suggested that this type is observed in all ages as a transition between multilocular and unilocular types.

## Conclusion

Human BATs in the posterior mediastinal descending para-aortic area were an organ with feeding vessels and sympathetic and parasympathetic innervation, could be histologically categorized into three subtypes: multilocular, paucilocular, and unilocular, was functionally affected by aging and ambient temperature, and was under different control compared with that of the supraclavicular area.

To the best of our knowledge, the mechanism underlying the differential functional regulation of supraclavicular and descending para-aortic BATs in the human thorax remains unclear. Further studies focusing on human descending para-aortic BATs may reveal how fat accumulates in BATs with aging and their role in sharing between the supraclavicular and descending para-aortic BATs and could establish an advanced strategy for a direct analysis of the association between the anatomical and functional properties of human BATs.
